# Validation of a predictive model for successful vaginal birth after cesarean section

**DOI:** 10.25100/cm.v50i1.2521

**Published:** 2019-03-30

**Authors:** Javier Enrique Fonseca, Juliana Lucía Rodriguez, Durley Maya Salazar

**Affiliations:** 1 Universidad del Valle, Facultad de Salud, Escuela de Medicina. Cali, Colombia; 2 Hospital Universitario del Valle Evaristo García, Cali, Colombia

**Keywords:** Vaginal birth after cesarean, cohort study, validation study, repeat cesarean section, obstetric delivery, trial of labor, labor presentation, cervical dilatation, Parto vaginal después de cesárea, estudio de cohorte, estudio de validación, cesárea repetida, parto obstétrico, trabajo de parto, presentación en trabajo de parto, dilatación cervical

## Abstract

**Introduction:**

A strategy for reducing the number of cesarean sections is to allow vaginal delivery after cesarean section.

**Objective:**

To validate two predictive models, Metz and Grobman, for successful vaginal delivery after a cesarean section.

**Methods:**

Retrospective cohort study involving women with previous history of a previous segmental cesarean section, single pregnancy ≥37 weeks and cephalic presentation. The proportion of vaginal delivery in all pregnant women was determined, and it was compared with those (women) with successful delivery after cesarean section. Then, there were elaborated the models, and their predictive capacity was determined by curve-receiver-operator.

**Results:**

The proportion of successful delivery in pregnant women with a previous cesarean section and indication of vaginal delivery was 85.64%. The observed proportion of birth for each decile predicted in the Grobman model was less than 15%, except for the 91-100% decile, where it was 64.09%; the area under the curve was 0.95. For the Metz model, the actual successful delivery rate was lower than predicted in scores between 4 and 14, and within expected for a score between 15 and 23; the area under the curve was 0.94.

**Conclusions:**

The vaginal delivery rate after cesarean was lower than expected according to the predictive models of Grobman and Metz. The implementation of these models in a prospective way can lead to a higher rate of successful birth.

## Resumen

**Introducción:**

Una estrategia de reducción del número de cesáreas es permitir el parto vaginal después de cesárea.

**Objetivo:**

Validar dos modelos predictivos, Metz y Grobman, para el parto vaginal exitoso después de una cesárea.

**Métodos:**

Estudio de cohorte retrospectivo con mujeres con antecedente de una cesárea segmentaria previa, embarazo único ≥37 semanas y presentación cefálica . Se determinó la proporción de parto vaginal en todas las gestantes y se comparó con aquellas con parto exitoso después de cesárea, se elaboró los modelos y se determinó la capacidad predictiva de ellos mediante curva-receptor-operador.

**Resultados:**

La proporción de parto exitoso en gestantes con cesárea previa e indicación de parto vaginal fue 85.64%. La proporción de parto observado para cada decil predicho en el modelo de Grobman fue inferior al 15%, excepto para el decil 91-100%, en el que fue 64.09%, el área bajo la curva fue 0.95. Para el modelo de Metz, la proporción de parto exitoso real fue menor a lo predicho en puntajes entre 4-14 y dentro de lo esperado para puntaje entre 15-23, con un área bajo la curva de 0.94.

**Conclusiones:**

La tasa de parto vaginal después de cesárea fue menor a lo esperado de acuerdo a los modelos predictivos de Grobman y Metz. La implementación de estos modelos en forma prospectiva puede llevar una mayor tasa de parto exitoso.

## Introduction 

Cesarean section (C-section) is one of the most common surgical procedures performed in women, especially in developed countries. In 1985, the World Health Organization (WHO) recommended that the rate for cesarean rate should not exceed 10-15% of total births ^1^. However, there’s an increasing tendency (to perform this procedure); by the 1990s in the United States, the percentage of C-sections increased up to 50% (20.7% in 1996 and 31.1% in 2006) ^2^. In 2011, one in three women had a cesarean delivery ^3^. If this trend continues, by 2020, it will be reached a rate of 56.2% for C-sections ^4^. In Colombia, the current rate is 45.7%; while in 1998, it was 24.9% ^5^. At *Hospital Universitario del Valle*, the proportion of C-sections in the last five years ranged between 27% and 29%.

The most common indications in the world for a primary cesarean include: stationary labor, altered or indeterminate fetal monitoring, anomalous fetal presentation and multiple gestation; corresponding the first two ones to more than half ^6^. Up to 30.9% of iterative C-sections are indicated by a previous C-section.

At the beginning of the 20th century, there prevailed the concept of "once a cesarean, always a cesarean" ^7^; however, by 1982, the American College of Obstetrics and Gynecology (ACOG) made recommendations on VBAC (Vaginal Birth after Cesarean), considering it like an "Acceptable option", and even proposing in 1995 that "all women should be taken to VBAC in the absence of a medical or obstetric contraindication" ^8^. In 2010, the National Institutes of Health of the United States (NHI), developed a consensus panel to address the practice of TOLAC (Trial of Labor after Cesarean) in the USA, concluding that "it is a reasonable option for many pregnant women." Pregnant woman should be guaranteed support and counseling in making the decision to try VBAC versus being taken to a repeated C-section.

Between 1996 and 2010, VBAC was reduced from 28% to 8% ^9^. Although ACOG reaffirmed the TOLAC opportunity in 2010, a study showed that only 52% of gynecologists offered VBAC in private practice ^10^ ^,^ ^11^.

In 2007, Grobman ^12^ developed a prediction nomogram for the success of VBAC based on factors available at the first prenatal check-up: Maternal age, BMI, ethnic group, previous vaginal delivery, successful vaginal delivery after C-section (the occurrence of a VBAC), and recurrence of the indication of primary cesarean section, all of which had an adequate predictive value.

In 2009, Grobman ^13^ included several factors at the time of admission to the delivery room: BMI at delivery, preeclampsia, gestational age at birth, cervical dilatation, effacement, stage and induction of labor, achieving a better performance of the model.

Recently, Metz *et al*. ^14^, created and validated a prediction model using variables (that were) available at the time of admission. The Bishop index, adding to it points for vaginal birth history, age <35 years, absence of a recurrent indication for C-section and BMI <30, generated a probability of successful VBAC higher than 85% in pregnant women with a score >16. When comparing this model with those previously described by Grobman ^12^ (factors at the first CPN and at the time of admission), the model developed by Metz *et al*. ^14^, presented the best performance.

*Hospital Universitario del Valle* is an institution that provides services for a population of high obstetric risk; an average of 7,500 births are attended every year, of which, 28-30% are by C-section. Currently, it is considered that the route of termination of a pregnancy, after cesarean delivery, is by vaginal delivery, but no predictive model is applied, and the decision depends mainly on the pelvic assessment and the Bishop index upon admission. For those reasons, the main objective of this study was to validate two predictive models, those by Metz and Grobman, for successful vaginal delivery after C-section (VBAC) in a pregnant population with previous C-section that enter to the Hospital for delivery; and to describe the maternal and fetal morbidity associated with vaginal delivery after C-section.

## Materials and Methods

Retrospective cohort study, in women who were pregnant for 37 weeks or longer, with a previous C-section and who were admitted to HUV during the study period (January-2009 to December-2013); there were included all pregnant women with a only previous cesarean procedure, pregnancy of 37 weeks or longer, and fetus in cephalic presentation at the time of defining the end of pregnancy; there were excluded patients with previous corporal C-section documented in the clinical history, previous uterine surgery (myomectomy, uterine rupture) or fetal death before the moment of defining the route of termination of pregnancy, as well as patients with inadequate pelvis.

The evaluated clinical variables were those included in the Grobman and Metz models, that is: ***Antepartum Variables***: maternal age (years), body mass index (Kg/m^2^), ethnicity, prior vaginal delivery, the occurrence of VBAC and recurring indication for C-section; ***Variables determined at admission to labor***: gestational age (complete weeks from the last reliable menstruation date or from the first available ultrasound), cervical dilatation, effacement, station, position and cervical consistency, induction of labor, cervical ripening, state of the membranes upon admission; and ***Maternal-fetal complications***: uterine rupture, vaginal tear, uterine hypotonia, postpartum hemorrhage, bladder injury, pelvic or abdominal organ injury, maternal infection (endometritis, panmetritis, episiotomy infection, surgical wound infection), weight of the newborn, Apgar score at minute and at 5 minutes, vitality at birth, neonatal complications (shoulder dystocia, clavicle fracture, ICU admission, death). In addition, **the outcome variable** was **the mode of delivery**: vaginal (spontaneous or instrumented) or C-section.

The information was collected in a Data Collection Format designed for that purpose and then exported to the Stata 10^®^ program for analysis. For quality control, 20% of the medical records were reviewed and double typing was done. In case of discrepancies, the database was confronted with the data collection formats. The study was approved by the ethics committee of *Universidad del Valle* and HUV.

### Sample size and analysis

The sample size was determined according to the recommendation of Harrel ^15^ for validating a multivariate prediction model, according to which no less than 10 desired results are required (successful delivery after C-section) for each variable included in the prediction model. In the case of the Grobman and Metz models that included antepartum and intra-partum variables, 13 variables were considered, which required 130 successful vaginal births. Taking into account an estimated success rate of delivery of 50% and a potential loss of 30% of information in the records in the clinical history, it was obtained a final sample size of 338 patients.

The cumulative incidence of vaginal delivery after C-section was determined by the equation: patients with successful vaginal delivery (VBAC)/total patients with a previous C-section during the observation period. The proportion of deliveries in patients allowed TOLAC was calculated with the equation: patients with successful vaginal delivery (VBAC)/patients with previous C-section in whom labor was attempted. 

For clinical characteristics at entering (the study), it was carried out a univariate analysis of the different independent variables according to the level of measurement of the variables; for continuous variables, there were used the Student's t-test and the Wilcoxon rank-sum test according to the distribution of the variables; and the Chi-squared test (chi^**2**^ ) or the Fisher's exact test for categorical data, according to the expected values ​​in each one of the cells. Subsequently, a multivariate analysis was carried out using logistic regression, including in the model those variables that in the univariate analysis obtained a value of *p* <0.2 or those that theoretically are considered predictive potentials, that is, those included in the models to be validated.

Maternal and fetal morbidity are presented as absolute and relative frequencies. The comparison of complications was performed using chi^**2**^ or Fisher's exact test, according to the expected values ​​in each one of the cells.

To determine the predictive capacity of the Grobman model in the population of HUV, the predicted probability was determined by using the equation previously published by Grobman ^13^ for each one of the pregnant women.

Delivery prediction = exp (w)/[1 + exp (w)] 

where w = 7.059 - 0.037 (age) - 0.44 (BMI) -0.46 (Afro-Americans) - 0.761 (Hispanic population) + 0.955 (any vaginal delivery before C-section) + 0.851 (vaginal delivery after C-section) - 0.655 (recurrent indication for C-section) - 0.109 (estimated gestational age at delivery) - 0.499 (hypertensive disease of pregnancy) + 0.044 (effacement) + 0.109 (dilatation ) + 0.082 (station) - 0.452 (induction of labor).

This prediction probability was divided into 10 deciles (0-10%, 11-20%, 21-30%, etc.); in each category, it was determined the actual (95%) proportion of success (that was) found. The predictive capacity of the model was established by calculating the area under the curve (AUC) of the operator receiver curve (ROC). The area under the curve was determined non-parametrically (in a non-parametric way), using the trapezoidal rule. Finally, a graphical comparison was made of the observed values ​​in the midpoints of each decile, in relation to the predicted values.

For the Metz model, the predicted probabilities were calculated using the points assigned to each variable of the model, (which were) previously published by the author ^14^, for each pregnant woman; subsequently, it was determined the actual probability (95% CI) of success. For the determination of the predictive capacity, it was constructed a logistic regression with the model variables, and the AUC of the operator receiver curve (ROC) was subsequently calculated. The area under the curve was determined non-parametrically, using the trapezoidal rule. For both models, sensitivity (S), specificity (E), positive and negative likelihood ratios (RV^+^ and RV^-^) were determined.

## Results

**Figure 1:**
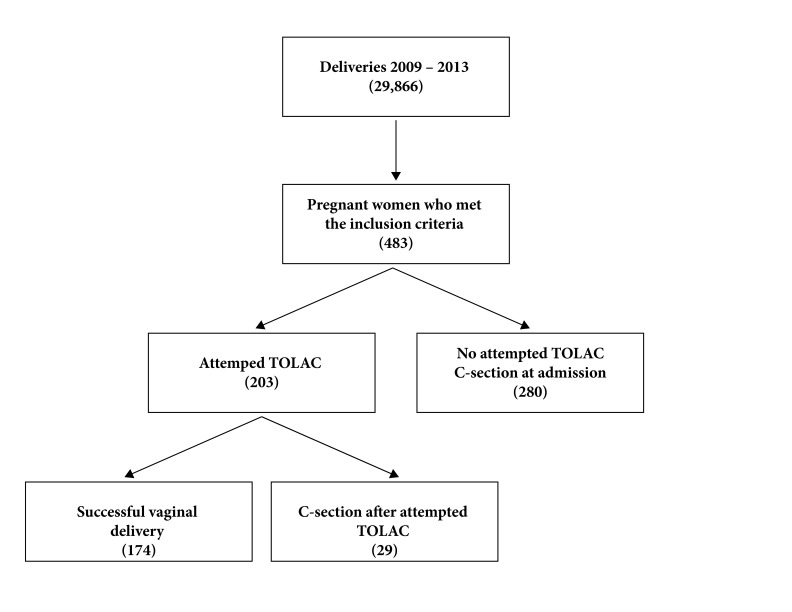
Flowchart of the patients in the VBAC recruiting process

During the years 2009 to 2013, there were 29,866 births in the HUV, of which 8,572 were via C-section, for a proportion of C-sections of 28.7%. In the same period, there were 483 pregnant women who met the inclusion criteria of the study and who did not present exclusion criteria, of which 203 (42.0%) were offered TOLAC, and 280 were scheduled for C-section from admission (Fig. 1). The characteristics of pregnant women for entering the study are presented in Table 1 and, according to the expected, there are statistically significant differences in most of them, except for maternal age, gestational age, proportion of patients with delivery before the initial C-section, and proportion of diabetes. 

**Table 1:** Maternal characteristics of the pregnant women according to attempted TOLAC or C-section

Characteristics	Vaginal delivery n: 203	C-section n: 280	*p*
Age* (years)	24 (21-29)	25 (22-30)	§0.034
Parity n (%)			
Nullipara	141 (69.46)	237 (84.64)	†0.000
Multipara	59 (29.06)	43 (15.36)	
Grand Multipara	3 (1.48)	0 (0.00)	
Birth before C-section n (%)	35 (17.24)	27 (9.64)	‡0.073
Birth after C-section n (%)	36 (17.73)	22 (7.86)	‡0.000
Race n (%)	196	277	
Black	56 (28.57)	105 (37.91)	†0.045
Other race	137 (69.90)	171 (61.73)	
Indigenous population	3 (1.53)	1 (0.36)	
BMI (Kg/m^2^)	24.15 (22.19 - 28.84)	26.11 (23.38 - 29.65)	§0.000
Gestational age* (ss)	39 (38 - 40)	39 (38 - 40)	§0.197
Dilatation (Value in Bishop’s score) n (%)			
0	3 (1,48)	159 (56.78)	†0.000
1	40 (19,70)	105 (37.50)	
2	75 (36.95)	12 (4.29)	
3	85 (41.87)	4 (1.43)	
Effacement (Value in Bishop’s score) n (%)			
0	3 (1.48)	189 (67.50)	†0.000
1	10 (4.93)	51 (18.21)	
2	69 (33.99)	31 (11.07)	
3	121 (59.61)	9 (3.22)	
Station (Value in Bishop’s score) n (%)			
0	13 (6,40)	212 (75.72)	†0.000
1	81 (39.90)	51 (18.21)	
2	108 (53.21)	17 (6.07)	
3	1 (0.49)	0 (0.00)	
Position (Value in Bishop’s score) n (%)	202	280	
0	13 (6.44)	227 (81.07)	‡0.000
1	91 (45.05)	43 (15.36)	
2	98 (48.51)	10 (3.57)	
Consistency (Value in Bishop’s score)	202	280	
0	0 (0.00)	186 (66.43)	†0.000
1	63 (31.19)	56 (20.00)	
2	139 (68.81)	38 (13.57)	
Bishop’s score at admission n (%)			
< 6	30 (14.78)	260 (92.86)	‡0.000
≥ 7	173 (85.22)	20 (7.14)	
Membranes status at admission n (%)	202	280	
Intact	141 (69.80)	258 (92.14)	‡0.000
Ruptured	61 (30.20)	22 (7.86)	
Diabetes n (%)	10 (4.93)	22 (7.86)	‡0.181
Pre-eclampsia n (%)	24 (11.82)	80 (28.57)	‡0.000

* Median (RIQ),

§ Wilcoxon rank-sum test,

† Fisher's exact test,

‡ Chi^2^,

BMI: Body Mass Index

The number of deliveries was 174 (171 spontaneous and 3 instrumented), for a proportion of deliveries in pregnant women with a previous C-section of 36.0%. The majority of patients who had a delivery (86.2%) entered with Bishop's index ≥7, while only 13.9% of the pregnant women who underwent C-section were admitted with a favorable index of Bishop for induction. The median of the Bishop index (RIQ) at admission was 8 (0-4) for pregnant women with C-section and 10 (8-11) for those who completed delivery.

Of the 203 pregnant women subjected to TOLAC, 174 (85.7%) had a delivery. The characteristics of admission are presented in Table 2, in which significant differences are observed in the proportion of labor after C-section and cervical dilatation at admission, being higher in both cases in the group of successful vaginal delivery. 

**Table 2:** Characteristics at admission of pregnant women with a previous C-section, submitted to TOLAC

	Vaginal delivery (174)	C-Section (29)	*p*
Age* (years)	24 (21 - 28)	24 (22 - 29)	§0.320
Parity n (%)			
Nullipara	118 (67.82)	23 (79.31)	†0.550
Multipara			
53 (30.46)	6 (20.69)		
Grand Multipara
3 (1.72)	0 (0.00)
Birth before C-section n (%)	30 (17.24)	5 (17.24)	†0.990
Birth after C-section n (%)	35 (20.11)	1 (3.45)	†0.029
Race n (%)	169	27	
Black	48 (28.40)	8 (29.63)	†1.000
Other race	118 (69.82)	19 (70.37)	
Indigenous population	3 (1.78)	0 (0.00)	
BMC^*^ (Kg/M^2^)	24.45 (22.19 - 26.67)	25.24 (22.22 - 28.84)	§0.328
Gestational age^*^ (weeks)	39 (38 - 40)	39 (38 - 40)	§0.450
Dilatation (Value in Bishop’s score) n (%)			
0	1 (0.57)	2 (6.90)	†0.007
1	32 (18.39)	8 (27.59)	
2	62 (35.63)	13 (44.83)	
3	79 (45.40)	6 (20.69)	
Effacement (Value in Bishop’s score) n (%)			
0	3 (1.72)	0 (0.00)	†0.392
1	7 (4.02)	3 (10.34)	
2	58 (33.33)	11 (37.93)	
3	106 (60.92)	15 (51.42)	
Station (Value in Bishop’s score) n (%)** **			
0	7 (4.02)	6 (20.69)	†0.010
1	69 (39.66)	12 (41.38)	
2	97 (55.75)	11 (37.93)	
3	1 (0.57)	0 (0.00)	
Position (Value in Bishop’s score)	173	29	** **
0	13 (7.51)	0 (0.00)	†0.204
1	74 (42.77)	17 (58.62)	
2	86 (49.71)	12 (41.38)	
Consistency (Value in Bishop’s score)	173	29	** **
0	0 (0.00)	0 (0.00)	†0.261
1	52 (30,06)	11 (37.93)	
2	121 (69.99)	18 (62.07)	
Bishop’s score at admission n (%)			
< 6	24 (13.79)	6 (20.69)	†0.293
≥ 7	150 (86.21)	23 (79.31)	
Membranes status at admission n (%)	173	29	
Intact	118 (68.21)	23 (79.31)	‡0.434
Ruptured	55 (31.79)	6 (20.69)	
Diabetes n (%)	8 (4.62)	2 (6.90)	†0.805
Pre-eclampsia n (%)	20 (11.49)	4 (13.79)	†0.731

* Median (RIQ),

§ Wilcoxon rank-sum test,

† Fisher's exact test,

‡ Chi^2^,

BMI: Body Mass Index

The main cause of an iterative C-section was a Bishop’s index unfavorable to admission in 179 (58.1%) pregnant women, followed by cephalic-pelvic disproportion in 30 (9.7%) patients and suspicion of fetal macrosomia in 20 (6.5%) patients. 9 (2.9%) C-sections were performed due to unsatisfactory fetal status, with two newborns with Apgar less than 7 at 5 minutes. Nine C-sections were performed due to imminence of uterine rupture; however, the uterus did not present solution of continuity in any of them.

The real probability of VBAC for each decile of success probability of the Grobman model was lower than expected, being less than 20% in all deciles; except in the 91-100% decile, where the proportion of VBAC was 64.1% (Table 3). The number of births expected according to the probabilities predicted using the midpoint of each decile is 366, for an expected proportion of 75.0%, much higher than that found in the HUV, with a proportion of 36.0%. 

**Table 3:** Predicted and observed probabilities of successful vaginal delivery in the HUV, according to the Grobman model

Predicted probability VBAC (%)	Number of patients (n)	Attended births	Observed probability VBAC (%)	CI 95%
0-10	0		n/a	NA
11-20	3	0	0	NA
21-30	19	0	0	NA
31-40	31	1	3.23	(-3.11 - 9.56)
41-50	55	1	1.82	(-1.75 - 5.39)
51-60	39	1	2.56	(-2.4 - 7.60)
61-70	22	1	4.55	(-4.39 - 13.48)
71-80	26	0	0	NA
81-90	29	4	13.79	(0.99 - 26.60)
91-100	259	166	64.09	(58.22 - 69.96)
Total	483	174	36.02	

VBAC: Successful vaginal delivery

NA: It does not apply

For a score lower than 14 in the Metz model, the real probability of VBAC was lower than predicted; in contrast, for scores ≥14, the real probabilities were higher than 65%, without significant differences in relation to the predicted ones (Table 4). The number of births expected according to the predicted probabilities is 267, for an expected proportion of 55.4%, much higher than the one observed for 174 births.

**Table 4:** Predicted and real probabilities of successful vaginal delivery in the HUV, according to the Metz model

Metz score	Number of patients (n)	Predicted probability VBAC (%)	Observed probability (%)	CI 95%
4	38	11.7	0	NA
5	29	14.7	0	NA
6	16	19	0	NA
7	68	24.7	0	NA
8	27	31.9	0	NA
9	21	40.2	14.29	(-10.89 - 30.66)
10	22	49.1	4.55	(-4.39 - 13.48)
11	25	57.7	12	(-10.33 - 25-03)
12	17	65.6	17.65	(-10.80 - 36.73)
13	32	72.2	40.63	(23.29 - 57.96)
14	23	77.5	65.22	(45.27 - 85.17)
15	21	81.6	71.43	(51.58 - 91.28)
16	26	84.7	80.77	(65.28 - 96.26)
17	23	87	69.57	(50.29 - 88.84)
18	31	88.6	80.65	(66.47 - 94.82)
19	24	89.8	95.83	(87.64 - 100.00)
20	12	90.7	91.67	(75.29 - 108.04)
21	9	91.4	88.89	(67.06 - 110.72)
22	10	91.9	100	NA
23	8	92.3	87.5	(62.94 - 112.06)

VBAC: Successful vaginal delivery

NA: It does not apply

**Figure 2:**
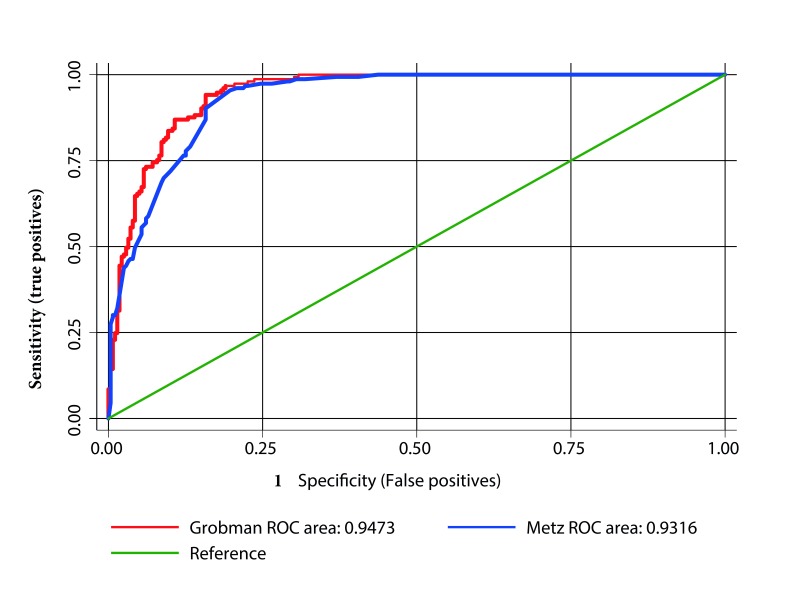
Receiver-operator curve for prediction of successful vaginal delivery with the Grobman and Metz models

The sensitivity, specificity and likelihood ratios of the Grobman and Metz model are presented in Table 5. Both models presented an area under the curve greater than 0.90 (Fig. 2) without significant differences between them (*p*= 0.38).

**Table 5:** Performance of predictive models of successful vaginal delivery after C-section

Characteristics of the model	Grobman^*^	Metz^*^	Grobman^**^	Metz^**^
Sensitivity (%)	83.55	78.16	83.91	75.29
Specificity (%)	89.93	88.64	89.97	89.64
LR+	8.30	6.88	8.36	7.27
LR-	0.18	0.25	0.18	0.28
Area under the curve (IC 95%)	0.947 (0.928-0.966)	0.938 (0.910-0.954)	0.943 (0.922-0.963)	0.929 (0.906-0.951)

* Model including all the variables included by the author

** Model including only variables that contribute to the model validated in the HUV.

LR: Likelihood Ratio

In Table 5, there are presented the sensitivity, specificity and likelihood ratios of the Grobman and Metz models, with only the variables that contributed to the prediction of the study, without significant differences in the areas under the curve with the original models (*p*= 0.09 for both models); however, with a difference between them (*p*= 0.009): a better performance for the Grobman model.

After selecting from the Grobman model only the variables that contribute to the logistic regression model for the pregnant women of the study, there persisted for our population the antecedent of delivery after C-section, effacement, station, dilatation at admission to the delivery service, and the presence of a hypertensive disorder. The association between these variables and the opportunity of delivery is presented in Table 6. In the Metz model, the only variable that persisted was the Bishop’s index (OR: 1.82 CI 95%: 1.64-2.01).

**Table 6:** Opportunity of successful vaginal delivery in pregnant women

Characteristic	OR (CI 95%)	ORa (CI 95%)
Birth after C-section	1.57 (0.94 - 2.60)	1.32 (0.82 - 2.12)
Dilatation	2.8 (2.33 - 3.35)	1.73 (1.39 - 2.17)
Effacement	1.08 (1.06 - 1.09)	1.01 (1.00 - 1.03)
Station	6.78 (4.90 - 9.38)	3.21 (1.95 - 5.28)
HTA	0.35 (0.20 - 0.59)	0.28 (0.11 - 0.71)
Induction	6.84 (3.68 - 12.71)	3.76 (1.70 - 8.35)

OR: odd ratio OR_a_: adjusted odd ratio

CI 95%: confidence interval 95%

HTA: Chronic arterial hypertension

Table 7 shows the more frequent maternal complications in the birthing group, with significant differences (*p*= 0.00); however, when discarding vaginal tears, which are an exclusive complication of labor, these differences disappear (*p*= 0.07).

**Table 7:** Maternal complications, characteristics and neonatal complications, according to birth pathway

Characteristic	Birth (n= 174)	C-section ( n= 309)	*p*
Maternal complications n (%)			
Vaginal tear III o IV	7 (4.02)	0	0.001
Post-partum hemorrhage	10 (5.75)	4 (1.29)	0.009
Some complication	15 (8.62)	5 (1.62)	0.000
Characteristics and neonatal complications			
Weight* (g)	3,065 (2,810- 3,320)	3,245 (2,900- 3,555)	0.0001
Sex n (%)			
Male	76 (43.68)	169 (54.69)	
Female	98 (56.32)	139 (44.98)	
Apgar at 5 minutes n (%)			
≤ 7	0 (0.0)	5 (1.4)	0.302
> 7	174 (100.0)	304 (93.4)	
Mechanical ventilation n (%)			
Yes	1 (0.6)	3 (1.0)	1.00
No	173 (99.4)	305 (98.7)	
Shoulder dystocia n (%)			
Yes	2 (1.2)	0 (0.0)	0.15

## Discussion

It was found in the present study that for both models, the observed probability of successful vaginal delivery after C-section was lower than predicted. In the case of the Grobman model for predicted probabilities lower than 90%, the observed ones were less than 20%; and for the Metz model for predicted probabilities lower than 70% (score lower than 13), the observed probabilities were less than 40%. This is possibly due to the fact that these models are not applied at HUV, and the delivery decision is based exclusively on medical criteria, depending on the Bishop’s index on admission, which was reflected in the fact that the delivery attempt (TOLAC) was made in only 42.0% of pregnant women.

Despite the above, the performance of both models was adequate, with areas under the curve higher than 0.90, a sensitivity of around 80%, and a false positive rate close to 10%, without significant differences between the two models (*p*= 0.38).

In 3,113 patients from 30 hospitals in Canada, subjected to TOLAC applying the Grobman model, Chaillet ^16^ found an AUC of 0.72 (95% CI: 0.70-0.74), lower than the yield in the present study; and in a cohort of 502 pregnant women, of which 262 (52.2%) had successful vaginal delivery, Constantine ^17^ found a similar AUC, 0.70 (95% CI: 0.65-0.74). Possibly this is because in these studies, only maternal characteristics were included in the model obtained in the first prenatal control, excluding variables at the moment of taking the decision to terminate the pregnancy, such as the characteristics of the cervix; and additionally, only 18% of the pregnant women in the study of Chaillet ^16^ and 10.76% in the study by Constantine ^17^ entered with a predicted probability greater than 90%; while in this investigation, 259 of 483 pregnant women (53%) entered with a probability of success greater than 90%, which possibly explains the higher performance of the model of Grobman in our investigation.

Not all the variables of the Grobman model contributed to the prediction of VBAC in the validation of the model in our study; only some cervical changes persisted at admission (dilatation, effacement and station), the antecedent of a birth after C-section and the presence of hypertensive disorder, which decreased the chance of VBAC; while for the Metz model, the only variable that persisted was Bishop's index at admission, which may be explained for being the cervical changes at the moment of pregnant woman's admission, a characteristic that determines the attempt of TOLAC.

In pregnant women undergoing TOLAC, there were found a delivery rate of 85.7% and a C-section of 14.3%, lower than the proportion of C-sections recorded in the HUV and in the same period, which was between 25-30%. This shows the adequate selection of pregnant women with a previous C-section undergoing TOLAC, based on cervical changes. However, we believe that the application of a predictive model in clinical practice can potentially increase the overall success rate, which in our case was only 36.0%. If TOLAC had been attempted in those pregnant women with a predicted probability equal to or greater than 50%, according to the Grobman model, TOLAC should have been attempted in 77.6% of the pregnant women and not only in 42.0%; while for the Metz model, the attempt of TOLAC should have occurred in 54.2% of the cases. 

The high rate of C-sections worldwide and nationally ^3^ ^,^ ^5^ ^,^ ^18^ ^,^ ^19^, leads to the need for strategies in the search to reduce their number; and it is recognized that the most important thing is to avoid primary C-section; however, with the increase of these, iterative C-section has become one of the first causes of C-section; for this reason, the identification of factors or maternal characteristics that select pregnant candidates for VBAC is a strategy that can potentially contribute to this objective. This and previous studies ^12^ ^,^ ^13^ show that cervical changes are the most important factors for the prediction of a successful birth after C-section; however, considering other variables such as the antecedent of a birth after C-section or the application of some predictive model can potentially lead to an increase in the number of pregnant women with a previous C-section in whom TOLAC is attempted.

One of the most important concerns for medical personnel is the risk of maternal complications such as uterine rupture and neonatal complications such as fetal death or neonatal asphyxia in patients undergoing delivery after C-section. In the present study, there were found no significant differences for 5 years between the pregnant women taken to C-section or delivery; however, there were more cases of postpartum hemorrhage in cases of vaginal delivery (5.75 vs. 1.29%), but in no case was there a need for hysterectomy within the management, or maternal death. With regard to newborns, no differences were found in the Apgar score at 5 minutes, or in the need for mechanical ventilation. Currently, there is no universally accepted discriminatory point regarding the predicted probability of success that is related to lower morbidity; however, apparently a probability of minimum success of 60-70% has equal or less probability of maternal complications for pregnant women subjected to TOLAC in relation to pregnant women taken to a repeated C-section ^20^ ^,^ ^21^, especially in pregnant women with a history of a previous birth.

The limitations of this study are those of the retrospective studies, based solely on the collection of information in the clinical history; however, there occurred missing data in less than 1% of the variables. Because of the retrospective nature, the decisions of delivery attempt depended on the criterion of the attending physician, and not on a previously standardized protocol, which may explain the low proportion of births (36.0%) in the population studied.

Most of the variables included in the Grobman and Metz models do not contribute to the final model of HUV, which may also be due to the retrospective nature of the study, since neither race, maternal age or antecedent of childbirth are considered as potential characteristics that modify the probability of delivery; currently, in pregnant women with unfavorable Bishop’s index, cervical ripening is not attempted with potentially useful alternatives such as the use of cervical balloon or dilatation through the use of trans-cervical Foley catheter; therefore, a prospective study with the application of a predictive model and with the implementation of cervical non-pharmacological ripening techniques, would allow a better validation of the models. 

Taking into account the similar performance between both models and the ease of application of the Metz model, we consider this one to be the most likely to be implemented in most obstetrical services, without the requirement of access to software or programs for determining the probability of successful vaginal delivery after C-section.
