# Supplementary material for: Validation of a predictive model for successful vaginal birth after cesarean section
Source: Colomb Med (Cali). 2019 Mar 30;50(1):13–21. doi: 10.25100/cm.v50i1.2521 (PMC6536040; doi:10.25100/cm.v50i1.2521)
Supplement: Related texts

# Original article: Validación de modelos predictivos para parto vaginal exitoso después de cesárea

## Introducción 

La cesárea es uno de los procedimientos quirúrgicos más comunes que se realiza en mujeres, especialmente en países desarrollados. En 1985, la Organización Mundial de la Salud (OMS) recomendó que la tasa de cesárea no debía exceder el 10-15% del total de nacimientos ^1^. Sin embargo, su tendencia va en aumento; hacia los años 90 en Estados Unidos el porcentaje de cesáreas aumentó hasta el 50%, (20.7% en 1996 y 31.1% en 2006) ^2^. En 2011, una de cada tres mujeres tuvieron parto por cesárea ^3^. Si esta tendencia continua, para el año 2020 se alcanzará una tasa de cesárea de 56.2% ^4^. En Colombia la tasa actual es 45.7%, mientras en 1998 fue 24.9% ^5^. En el Hospital Universitario del Valle la proporción de cesáreas en el último quinquenio osciló entre 27-29%.

Las indicaciones más comunes en el mundo para una cesárea primaria incluyen: trabajo de parto estacionario, monitorias fetales alteradas o indeterminadas, presentación fetal anómala y gestación múltiple, las dos primeras corresponden a más de la mitad ^6^ y hasta un 30.9% de las cesáreas iterativas tienen como indicación una cesárea previa .

A principios del siglo XX, se tuvo el concepto “una vez cesárea, siempre cesárea” ^7^, sin embargo, hacia 1982 el Colegio Americano de Ginecología y Obstetricia (ACOG) planteó recomendaciones sobre VBAC (Vaginal Birth after Cesarean), considerándolo una “opción aceptable”, e incluso proponiendo en 1995 que “todas las mujeres deberían ser llevadas a VBAC en ausencia de contraindicación medica u obstétrica” ^8^. En el 2010, el Instituto Nacional de Salud de los Estados Unidos (NHI), desarrolló un panel de consenso para direccionar la práctica de TOLAC (Trial of Labor after Cesarean) en USA concluyendo que “es una opción razonable para muchas mujeres embarazadas", debiendo garantizarse a la gestante apoyo y consejería en la toma de decisión de intentar VBAC versus ser llevada a cesárea repetida. 

Entre 1996 y 2010 se encontró que la VBAC se redujo del 28% al 8% ^9^. A pesar que la ACOG reafirmo la oportunidad de TOLAC en 2010, un estudio demostró que solo el 52% de los ginecólogos ofrecian VBAC en la práctica privada ^10^^,^^11^. 

En 2007, Grobman ^12^ desarrollo un nomograma de predicción para el éxito de VBAC basado en factores disponibles al primer control prenatal: La edad materna, IMC, raza, parto vaginal previo. La indicación para una cesárea primaria recurrente fueron los elementos incluidos, con un buen poder predictivo. En el 2009 Grobman ^13^ incluyó varios factores al momento de la admisión a partos: IMC al parto, preeclampsia, edad gestacional al parto, dilatación cervical, borramiento, estación e inducción del parto, logrando un mejor rendimiento del modelo. 

Recientemente Metz *et al*. ^14^, crearon y validaron un modelo de predicción usando variables disponibles al tiempo de la admisión. El índice de Bishop, sumándole puntos por historia de parto vaginal, edad <35 años, ausencia de indicación recurrente de cesárea e IMC <30, generaron una probabilidad de VBAC exitoso, superior al 85% en las gestantes con un puntaje >16. Al compararse este modelo con los descritos previamente de Grobman (factores al primer CPN y al momento de la admisión), el de Metz present él mejor rendimiento. 

El Hospital Universitario del Valle es una institución que maneja una población de alto riesgo obstétrico, atiende un promedio 7,500 nacimientos al año, de los cuales, el 28-30% son por vía cesárea. Actualmente se considera que la vía de finalización del embarazo después de una cesárea es el parto vaginal pero no se aplica ningún modelo predictivo y la decisión depende fundamentalmente de la valoración pélvica y del índice de Bishop al ingreso, por lo cual el objetivo principal del estudio fue validar dos modelos predictivos, Metz y Grobman, para parto vaginal exitoso después de cesárea VBAC en la población gestante con cesárea previa que ingresan para parto al Hospital y describir la morbilidad materna y fetal asociada al parto vaginal después de cesárea. 

## Materiales y Métodos 

Estudio de cohorte retrospectivo, en gestantes de 37 o más semanas con una cesárea previa que ingresaron para atención al Hospital Universitario del Valle (HUV) durante el periodo de estudio (Enero-2009 a Diciembre-2013), se incluyeron todas las gestantes con una sola cesárea previa, embarazo de 37 semanas o más y feto en presentación cefálica al momento de definir la finalización del embarazo, se excluyeron pacientes con cesárea previa corporal documentada en la historia clínica, antecedente de cirugía uterina previa (miomectomía, ruptura uterina) o muerte fetal antes del momento de definición de la vía de finalización del embarazo y pacientes con pelvis inadecuada. 

Las variables clínicas evaluadas fueron las incluidas en los modelos de Grobman y Metz asi: ***variables anteparto;*** edad materna (años), Índice de masa corporal (Kg/m^2^), etnia, antecedente de parto vaginal previo a la cesárea y de parto vaginal después de cesárea, gravidez, causa de cesárea previa; ***variables determinadas a la admisión al trabajo de parto*** ; edad gestacional (semanas completas a partir de fecha de ultima menstruación confiable o partir de primera ecografía disponible), dilatación cervical, borramiento, estación, posición y consistencia cervical, inducción del trabajo de parto, maduración cervical, estado de las membranas al ingreso y adicionalmente ***complicaciones maternofetales*** como ruptura uterina, desgarro vaginal, hipotonía uterina, hemorragia posparto, lesión vesical, lesión de órganos pélvico o abdominales, infección materna (endometritis, panmetritis, infección de episiotomía, infección de herida quirúrgica), peso del recién nacido, apgar al minuto y a los 5 minutos, vitalidad al nacimiento, complicaciones neonatales (distocia de hombros, fractura de clavícula, Ingreso a UCI, muerte). Adicionalmente **la variable resultado fue modo de parto**, vaginal (espontáneo o instrumentado) o cesárea.

La información se recolectó en un Formato de Recolección de Datos diseñado para tal fin y se exportó al programa Stata 10 ® para su análisis. Para control de calidad se realizó la revisión del 20% de las historias clínicas y se hizo doble digitación. En caso de discrepancias se confrontó la base de datos con los formatos de recolección de datos. el estudio fue aprobado por el comité de ética de la Universidad del Valle y del Hospital Universitario del Valle.

### Tamaño de muestra y análisis

El tamaño de muestra se determinó según la recomendación de Harrel ^15^ para la validación de un modelo multivariado de predicción, según la cual se requieren no menos de 10 resultados deseados (parto exitoso después de cesárea) por cada variable incluida en el modelo de predicción. En el caso de los modelos de Grobman y de Metz que incluyeron variables anteparto e intraparto se consideraron 13 variables, por lo cual se requerían 130 partos vaginales exitosos. Teniendo en cuenta una proporción de éxito estimada de parto del 50% y una potencial pérdida del 30% de información en los registros en la historia clínica se obtuvo un tamaño de muestra final de 338 pacientes. 

Se determinó la Incidencia acumulada de parto vaginal después de cesárea mediante la ecuación: pacientes con parto vaginal exitoso (VBAC)/total pacientes con una cesárea previa en periodo de observación, la proporción de partos en pacientes que se permitió TOLAC se calculó con la ecuación: pacientes con parto vaginal exitoso (VBAC)/pacientes con cesárea previa en quienes se intentó parto.

Para las características de ingreso se realizó un análisis univariado de las diferentes variables independientes de acuerdo al nivel de medición de las variables; para las variables continuas se utilizó la prueba T de student y rango de suma de wilconxon de acuerdo a la distribución de las variable y para las categóricas chi^2^ o la prueba exacta de Fisher de acuerdo a los valores esperados en cada una de las celdas. Posteriormente se realizó un análisis multivariado mediante la regresión logística incluyendo en el modelo aquellas variables que en el análisis univariado obtuvieron un valor de *p* <0.2 o las que teóricamente se consideran potenciales predictores, es decir las incluidas en los modelos a validar. 

La morbilidad materna y fetal se presenta como frecuencias absolutas y relativas. Se realizó la comparación de las complicaciones mediante chi^2^ o prueba exacta de Fisher de acuerdo a los valores esperados en cada una de las celdas.

Para determinar la capacidad predictiva del modelo de Grobman en la población del Hospital Universitario del Valle, se determinó la probabilidad predicha mediante el uso de la ecuación previamente publicada por Grobman ^13^ para cada una de las gestantes.

Predicción de parto = exp(w)/[1+exp(w)] 

donde w= 7.059 - 0.037(edad) - 0.44(IMC) -0.46(raza negra-raza americana) - 0.761(raza hispana) + 0.955(cualquier parto vaginal antes de cesárea) + 0.851(parto vaginal después de cesárea) - 0.655(indicación recurrente de cesárea) - 0.109(edad gestacional estimada al parto) - 0.499(enfermedad hipertensiva del embarazo) + 0.044(borramiento) + 0.109(dilatación) + 0.082(estación) - 0.452(inducción del parto).

Esta probabilidad de predicción se fraccionó en 10 deciles (0-10%, 11-20%, 21-30%, etc), en cada categoría se determinó la proporción (IC 95%) real de éxito encontrada. La capacidad predictiva del modelo se estableció mediante el cálculo del área bajo la curva (AUC) de la curva receptor operador (ROC). El área bajo la curva se determinó de forma no paramétrica, utilizando la regla trapezoidal. Finalmente se realizó una comparación grafica de los valores observados en los puntos medios de cada decil en relación a los valores predichos. 

Para el modelo de Metz se calculó las probabilidades predichas utilizando los puntos asignados a cada variable del modelo, previamente publicados por el autor ^14^, para cada gestante y posteriormente se determinó la probabilidad real (IC 95%) de éxito encontrada. Para la determinación de la capacidad predictiva se construyó una regresión logística con las variables del modelo y posteriormente se calculó el AUC de la curva receptor operador (ROC). El área bajo la curva se determinó de forma no paramétrica, utilizando la regla trapezoidal. Para ambos modelos se determinó la sensibilidad (S), especificidad (E), razones de verosimilitud positiva y negativa (RV^+^ y RV^-^).

## Resultados

Durante los años 2009 a 2013 se presentaron en el HUV 29,866 nacimientos de los cuales 8,572 fueron vía cesárea, para una proporción de cesáreas del 28.7%. En el mismo periodo se presentaron 483 gestantes que cumplieron con los criterios de inclusión del estudio y sin criterios de exclusión, de las cuales a 203 (42.0%) se les ofreció TOLAC y a 280 se las programo para cesárea desde el ingreso (Fig. 1). Las características de ingreso de las gestantes se presentan en la Tabla 1 y de acuerdo a lo esperado hay diferencias estadísticamente significantes en la mayoría de ellas, excepto para edad materna, edad gestacional, proporción de pacientes con parto previo a la cesárea inicial y en la proporción de diabetes. 

**Figura 1:** Flujograma de las pacientes en el proceso de reclutamiento VBAC

**Tabla 1:** Características maternas de gestantes de acuerdo a intento de TOLAC o cesárea.

Características	Parto n: 203	Cesárea n: 280	*p*
Edad* (años)	24 (21-29)	25 (22-30)	§0.034
Paridad n(%)			
Nulípara	141 (69.46)	237 (84.64)	†0.000
Multípara	59 (29.06)	43 (15.36)	
Gran Multípara	3 (1.48)	0 (0.00)	
Parto previo cesárea n(%)	35 (17.24)	27 (9.64)	‡0.073
Parto después de cesárea n(%)	36 (17.73)	22 (7.86)	‡0.000
Raza n(%)	196	277	
Negra	56 (28.57)	105 (37.91)	†0.045
Otra raza	137 (69.90)	171 (61.73)	
Indigena	3 (1.53)	1 (0.36)	
IMC (Kg/m^2^)	24.15 (22.19 - 28.84)	26.11 (23.38 - 29.65)	§0.000
Edad Gestacional* (ss)	39 (38 - 40)	39 (38 - 40)	§0.197
Dilatación (valor en escala bishop) n(%)			
0	3 (1,48)	159 (56.78)	†0.000
1	40 (19,70)	105 (37.50)	
2	75 (36.95)	12 (4.29)	
3	85 (41.87)	4 (1.43)	
Borramiento (valor en escala bishop) n(%)			
0	3 (1.48)	189 (67.50)	†0.000
1	10 (4.93)	51 (18.21)	
2	69 (33.99)	31 (11.07)	
3	121 (59.61)	9 (3.22)	
Estación (valor en escala bishop) n(%)			
0	13 (6,40)	212 (75.72)	†0.000
1	81 (39.90)	51 (18.21)	
2	108 (53.21)	17 (6.07)	
3	1 (0.49)	0 (0.00)	
Posición (valor en escala bishop) n(%)	202	280	
0	13 (6.44)	227 (81.07)	‡0.000
1	91 (45.05)	43 (15.36)	
2	98 (48.51)	10 (3.57)	
Consistencia (valor en escala bishop)	202	280	
0	0 (0.00)	186 (66.43)	†0.000
1	63 (31.19)	56 (20.00)	
2	139 (68.81)	38 (13.57)	
Bishop al ingreso n(%)			
< 6	30 (14.78)	260 (92.86)	‡0.000
≥ 7	173 (85.22)	20 (7.14)	
Estado membranas al ingreso n(%)	202	280	
Integras	141 (69.80)	258 (92.14)	‡0.000
Rotas	61 (30.20)	22 (7.86)	
Diabetes n(%)	10 (4.93)	22 (7.86)	‡0.181
Preeclampsia n(%)	24 (11.82)	80 (28.57)	‡0.000

* Mediana (RIQ),

§ Rango de sumas de wilconxon,

† Prueba exacta de fisher,

‡ Chi2,

IMC: Índice de Masa Corporal

El número de partos fue de 174 (171 espontáneos y 3 instrumentados), para una proporción de partos en gestantes con una cesárea previa de 36.0%. La mayor parte de pacientes que tuvieron parto (86.2%) ingresaron con índice de Bishop ≥7, mientras solo 13.9% de las gestantes a quienes se les realizó cesárea, ingresaron con índice de Bishop favorable para inducción. La mediana del índice de Bishop (RIQ) al ingreso fue 8 (0-4) para las gestantes con cesárea y 10 (8-11) para las que finalizaron por parto. 

De las 203 gestantes sometidas a TOLAC, 174 (85.7%) tuvieron parto. Las características de ingreso se presentan en la Tabla 2, en la cual se observa diferencias significativas en la proporción de parto después de cesárea y en la dilatación cervical al ingreso, siendo mayores en ambos casos en el grupo de parto vaginal exitoso. 

**Tabla 2:** Características de ingreso de gestantes con cesárea previas sometidas a TOLAC

	Parto (174)	Cesárea (29)	*p*
Edad* (años)	24 (21 - 28)	24 (22 - 29)	§0.32
Paridad n(%)			
Nulipara	118 (67.82)	23 (79.31)	†0.550
53 (30.46)	6 (20.69)		
Multipara			
Gran Multipara
3 (1.72)	0 (0.00)
Parto previo cesarea n(%)	30 (17.24)	5 (17.24)	†0.99
Parto despues de cesarea n(%)	35 (20.11)	1 (3.45)	†0.029
Raza n(%)	169	27	
Negra	48 (28.40)	8 (29.63)	†1.000
Otra raza	118 (69.82)	19 (70.37)	
Indigena	3 (1.78)	0 (0.00)	
IMC^*^ (Kg/M^2^)	24.45 (22.19 - 26.67)	25.24 (22.22 - 28.84)	§0.328
Edad Gestacional^*^ (ss)	39 (38 - 40)	39 (38 - 40)	§0.450
Dilatacion (valor en escala bishop) n(%)			
0	1 (0.57)	2 (6.90)	†0.007
1	32 (18.39)	8 (27.59)	
2	62 (35.63)	13 (44.83)	
3	79 (45.40)	6 (20.69)	
Borramiento (valor en escala bishop) n(%)			
0	3 (1.72)	0 (0.00)	†0.392
1	7 (4.02)	3 (10.34)	
2	58 (33.33)	11 (37.93)	
3	106 (60.92)	15 (51.42)	
Estacion (valor en escala bishop) n(%)			
0	7 (4.02)	6 (20.69)	†0.010
1	69 (39.66)	12 (41.38)	
2	97 (55.75)	11 (37.93)	
3	1 (0.57)	0 (0.00)	
Posicion (valor en escala bishop)	173	29	** **
0	13 (7.51)	0 (0.00)	†0.204
1	74 (42.77)	17 (58.62)	
2	86 (49.71)	12 (41.38)	
Consistencia (valor en escala bishop)	173	29	** **
0	0 (0.00)	0 (0.00)	†0.261
1	52 (30,06)	11 (37.93)	
2	121 (69.99)	18 (62.07)	
Bishop al ingreso n(%)			
< 6	24 (13.79)	6 (20.69)	†0.293
≥ 7	150 (86.21)	23 (79.31)	
Estado membranas al ingreso n(%)	173	29	
Integras	118 (68.21)	23 (79.31)	‡0.434
Rotas	55 (31.79)	6 (20.69)	
Diabetes n(%)	8 (4.62)	2 (6.90)	†0.805
Preeclampsia n(%)	20 (11.49)	4 (13.79)	†0.731

* Mediana (RIQ),

§ Rango de sumas de wilconxon,

† Prueba exacta de fisher,

‡ Chi2,

IMC: Índice de Masa Corporal

La principal causa de cesárea iterativa fue un índice de Bishop desfavorable al ingreso en 179 (58.1%) gestantes, seguida por desproporción cefalopélvica en 30 (9.7%) pacientes y sospecha de macrosomía fetal en 20 (6.5%) pacientes. Se realizaron 9 (2.9%) cesáreas por estado fetal no satisfactorio, con dos recién nacidos con apgar menor de 7 a los 5 minutos. Nueve cesáreas se realizaron por inminencia de ruptura uterina, sin embargo, en todas ellas el útero no presento solución de continuidad.

La probabilidad real de VBAC para cada decil de probabilidad de éxito del modelo de Grobman fue menor a lo esperado, siendo inferior al 20% en todos los deciles, excepto en el decil 91-100%, donde la proporción de VBAC fue 64.1% (Tabla 3). El número de partos esperados de acuerdo a las probabilidades predichas utilizando el punto medio de cada decil es de 366, para una proporción esperada del 75.0%, mucho mayor a lo encontrado en el HUV con una proporción de 36.0%.

**Tabla 3:** Probabilidades predichas y observadas de parto vaginal exitoso de acuerdo al modelo de Grobman en el HUV

Probabilidad predicha VBAC (%)	Numero pacientes (n)	Partos atendidos	Probabilidad observada VBAC (%)	IC 95%
0 - 10	0		n/a	NA
11 - 20	3	0	0	NA
21 - 30	19	0	0	NA
31 - 40	31	1	3.23	(-3.11 - 9.56)
41 - 50	55	1	1.82	(-1.75 - 5.39)
51 - 60	39	1	2.56	(-2.4 - 7.60)
61 - 70	22	1	4.55	(-4.39 - 13.48)
71 - 80	26	0	0	NA
81 - 90	29	4	13.79	(0.99 - 26.60)
91 - 100	259	166	64.09	(58.22 - 69.96)
Total	483	174	36.02	

VBAC: Parto vaginal exitoso

NA:No aplica

Para el modelo de Metz en puntaje inferior a 14 la probabilidad real de VBAC fue menor a las predicha y para puntaje ≥14, las probabilidades reales fueron superiores a 65%, sin diferencias significativas en relación a las predichas (Tabla 4). El número de partos esperados de acuerdo a las probabilidades predichas es de 267, para una proporción esperada del 55.4%, mucho mayor a lo observado de 174 partos.

**Tabla 4:** Probabilidades predichas y reales de parto vaginal exitoso de acuerdo al modelo de Metz en el HUV

Puntaje Metz	Numero de pacientes (n)	Probabilidad predicha VBAC (%)	Probabilidad observada (%)	IC 95%
4	38	11.7	0	NA
5	29	14.7	0	NA
6	16	19	0	NA
7	68	24.7	0	NA
8	27	31.9	0	NA
9	21	40.2	14.29	(-10.89 - 30.66)
10	22	49.1	4.55	(-4.39 - 13.48)
11	25	57.7	12	(-10.33 - 25-03)
12	17	65.6	17.65	(-10.80 - 36.73)
13	32	72.2	40.63	(23.29 - 57.96)
14	23	77.5	65.22	(45.27 - 85.17)
15	21	81.6	71.43	(51.58 - 91.28)
16	26	84.7	80.77	(65.28 - 96.26)
17	23	87	69.57	(50.29 - 88.84)
18	31	88.6	80.65	(66.47 - 94.82)
19	24	89.8	95.83	(87.64 - 100.00)
20	12	90.7	91.67	(75.29 - 108.04)
21	9	91.4	88.89	(67.06 - 110.72)
22	10	91.9	100	NA
23	8	92.3	87.5	(62.94 - 112.06)

VBAC: Parto vaginal exitoso

NA:No aplica

La sensibilidad, especificidad y razones de verosimilitud del modelo de Grobman y de Metz se presentan en la Tabla 5. Ambos modelos presentaron un área bajo la curva superior a 0.90 (Fig. 2) sin diferencias significativas entre ellos (*p*= 0.38). 

**Figura 2:** Curva receptor-operador para predicción de parto vaginal exitoso con los modelos de Grobman y Metz

**Tabla 5:** Rendimiento de modelos de predicción de parto vaginal exitoso después de cesárea

Caracteristica del modelo	Grobman^*^	Metz^*^	Grobman^**^	Metz^**^
Sensibilidad (%)	83.55	78.16	83.91	75.29
Especificidad (%)	89.93	88.64	89.97	89.64
RV+	8.3	6.88	8.36	7.27
RV-	0.18	0.25	0.18	0.28
Area bajo curva (IC 95%)	0.947 (0.928-0.966)	0.938 (0.910-0.954)	0.943 (0.922-0.963)	0.929 (0.906-0.951)

* Modelo incluyendo todas las variables incluidas por el autor.

** Modelo incluyendo solo variables que aportan al modelo validado en el HUV. RV: Razon de verosimilitud

La sensibilidad, especificidad y razones de verosimilitud de los modelos de Grobman y Metz con solo las variables que aportaron a la predicción del estudio se presentan en la Tabla 5, sin diferencias significativas en las áreas bajo la curva con los modelos originales (*p*= 0.09 para ambos modelos), sin embargo, con diferencia entre ellos (*p*= 0.009) con un mejor rendimiento para el modelo de Grobman. 

Después de seleccionar del modelo de Grobman solo las variables que aportan al modelo de regresión logística para las gestantes del estudio, persistieron para nuestra población el antecedente de parto después de cesárea, el borramiento, estación, dilatación al ingreso al servicio de partos y la presencia de un trastorno hipertensivo. La asociación entre estas variables y la oportunidad de parto se presenta en la Tabla 6. En el modelo de Metz la única variable que persistió fue el índice Bishop (OR: 1.82 IC 95%: 1.64-2.01). 

**Tabla 6:** Oportunidad de parto vaginal exitoso en gestantes

Característica	OR (IC 95%)	ORa (IC 95%)
Parto después cesárea	1.57 (0.94 - 2.60)	1.32 (0.82 - 2.12)
Dilatación	2.8 (2.33 - 3.35)	1.73 (1.39 - 2.17)
Borramiento	1.08 (1.06 - 1.09)	1.01 (1.00 - 1.03)
Estación	6.78 (4.90 - 9.38)	3.21 (1.95 - 5.28)
HTA	0.35 (0.20 - 0.59)	0.28 (0.11 - 0.71)
Inducción	6.84 (3.68 - 12.71)	3.76 (1.70 - 8.35)

OR: odd ratio OR_a_: odd ratio ajustado

IC 95%: intervalo confianza 95%

HTA: Hipertension arterial crónica

En la Tabla 7 se presentan las complicaciones maternas las cuales fueron más frecuentes en el grupo de parto, con diferencias significativas (*p*= 0.00), sin embargo, al descartar los desgarros vaginales los cuales son una complicación exclusiva del parto, estas diferencias desaparecen (*p*= 0.07). 

**Tabla 7:** Complicaciones maternas, características y complicaciones neonatales de acuerdo a vía de nacimiento

Característica	Parto (n= 174)	Cesárea ( n= 309)	*p*
Complicaciones maternas n (%)			
Desgarro vaginal III o IV	7 (4.02)	0	0.001
Hemorragia postparto	10 (5.75)	4 (1.29)	0.009
Alguna complicación	15 (8.62)	5 (1.62)	0.000
Características y complicaciones neonatales			
Peso* (g)	3,065 (2,810- 3,320)	3,245 (2,900- 3,555)	0.0001
Sexo n (%)			
Femenino	98 (56.3)	139 (45.0)	
Apgar a los 5 minutos n(%)			
≤ 7	0 (0.0)	5 (1.4)	0.302
> 7	174 (100.0)	304 (93.4)	
Ventilación mecánica n(%)			
Si	1 (0.6)	3 (1.0)	1.00
No	173 (99.4)	305 (98.7)	
Distocia de hombro n(%)			
Si	2 (1.2)	0 (0.0)	0.15

* mediana (RIQ)

## Discusión 

En el presente estudio se encontró que para ambos modelos la probabilidad observada de parto vaginal exitoso después de cesárea fue menor a la predicha. En el caso del modelo de Grobman para probabilidades predichas inferiores al 90%, las observadas fueron menores al 20% y para el modelo de Metz para probabilidades predichas inferiores al 70% (puntaje inferior a 13), las probabilidades observadas fueron inferiores a 40%, esto se debe posiblemente a que en la institución no se aplican estos modelos y la decisión de parto se basa exclusivamente en el criterio médico, dependiendo del índice de Bishop al ingreso, lo cual se reflejó en que solo se realizó el intento de parto (TOLAC) en el 42.0% de las gestantes.

A pesar de lo anterior el rendimiento de ambos modelos fue adecuado con áreas bajo la curva superiores al 0.90, una sensibilidades alrededor del 80% y una tasa de falsos positivos cercana al 10%, sin diferencias significativas entre ambos modelos (*p*= 0.38). 

Chaillet ^16^ en 3,113 pacientes de 30 hospitales en Canadá, sometidas a TOLAC aplicando el modelo de Grobman, encontró un AUC de 0.72 (IC 95% 0.70-0.74), inferior al rendimiento en el presente estudio y Constantine ^17^ en una cohorte de 502 gestantes, de las cuales 262 (52.2%) tuvieron parto vaginal exitoso, encontró un AUC similar, 0.70 (IC 95% 0.65-0.74), esto posiblemente se debe a que en estos estudios solo se incluyeron en el modelo características maternas obtenidas en el primer control prenatal, excluyendo variables en el momento de toma de la decisión de finalización del embarazo como las características del cérvix y adicionalmente solo 18% de las gestantes en el estudio de Chaillet ^16^ y 10.76% en el estudio se Constantine ^17^, ingresaron con una probabilidad predicha mayor del 90%, mientras en esta investigación, 259 de 483 gestantes (53%) ingresaron con una probabilidad de éxito mayor a 90%, lo cual posiblemente explica el mayor rendimiento del modelo de Grobman en nuestra investigación.

No todas las variables del modelo de Grobman aportaron a la predicción de VBAC en la validacion del modelo en nuestro estudio, solo persistieron algunos cambios cervicales al ingreso (dilatación, borramiento y estación), el antecedente de un parto después de cesárea y la presencia de trastorno hipertensivo el cual disminuyó la oportunidad de VBAC, mientras para el modelo de Metz la única variable que persistió fue el índice de Bishop al ingreso, lo anterior posiblemente explicado por ser los cambios cervicales al ingreso de la gestante la característica que determina el intento de TOLAC.

En gestantes sometidas a TOLAC se encontró una proporción de parto del 85.7% y una proporción de cesárea del 14.3%, inferior a la proporción de cesárea registradas en el HUV y en el mismo periodo, la cual estuvo entre 25-30%. Esto muestra la adecuada selección de las gestantes con cesárea previa sometidas a TOLAC, basada en los cambios cervicales. Sin embargo consideramos que la aplicación de un modelo predictivo en la práctica clínica, potencialmente puede aumentar la proporción de éxito global que en nuestro caso fue de solo 36.0%. Si se hubiera intentado TOLAC en aquellas gestantes con una probabilidad predicha igual o superior al 50% según el modelo de Grobman se debió haber intentado TOLAC en el 77.6% de las gestantes y no solo en el 42.0%, mientras que para el modelo de Metz el intento de TOLAC debió ser en 54.2% de los casos.

La alta tasa de cesárea a nivel mundial y nacional ^3^^,^^5^^,^^18^^,^^19^, lleva a la necesidad de estrategias en la búsqueda de reducir su número y se reconoce que lo más importante es evitar la cesárea primaria, sin embargo, con el aumento de estas, la cesárea iterativa se ha convertido en una de las primeras causas de cesárea, por lo cual la identificación de factores o características maternas que seleccionen gestantes candidatas para VBAC es una estrategia que potencialmente puede contribuir a este objetivo. El presente estudio y previos ^12^^,^^13^ muestran que los cambios cervicales son los factores más importantes, sin embargo, el considerar otras variables como el antecedente de parto después de cesárea o la aplicación de algún modelo de predicción potencialmente puede llevar a un aumento del número de gestantes con cesárea previas en quienes se intenta TOLAC. 

Una de las preocupaciones más importantes en el personal médico es el riesgo de complicaciones maternas como la ruptura uterina y, neonatales como muerte fetal o asfixia neonatal, en pacientes sometidas a parto después de cesárea. En el presente estudio durante 5 años no se encontró diferencias significativas entre las gestantes llevadas a cesárea o a parto, no obstante, hubo más casos de hemorragia postparto en casos de parto vaginal (5.75 vs 1.29%), pero en ningún caso hubo necesidad de histerectomía dentro del manejo o muerte materna. Con respecto a los recién nacidos no se encontró diferencias en el apgar a los 5 minutos o en la necesidad de ventilación mecánica. No existe en la actualidad ningún punto discriminatorio universalmente aceptado respecto a la probabilidad predicha de éxito que se relaciona con menor morbilidad, sin embargo, aparentemente una probabilidad de éxito mínima del 60 -70%, tiene igual o menor probabilidad de complicaciones maternas para gestantes sometidas a TOLAC en relación a gestantes llevadas a una cesárea a repetición ^20^^,^^21^, especialmente en gestantes con antecedente de un parto previo. 

Las limitaciones del estudio son las propias de los estudios retrospectivos, basado únicamente en la recolección de información en la historia clínica, sin embargo, se presentaron datos faltantes en menos del 1% de las variables. Por la naturaleza retrospectiva, las decisiones de intento de parto dependieron del criterio del médico tratante y no de un protocolo previamente estandarizado, lo cual puede explicar la baja proporción de partos (36.0%) en la población estudiada. 

La mayoría de variables incluidas en los modelos de Grobman y de Metz, no aportan al modelo final del Hospital Universitario del Valle, lo cual puede ser debido también a la característica retrospectiva del estudio, puesto que ni la raza, ni la edad materna o el antecedente de parto se consideran como características potenciales que modifiquen la probabilidad de parto y en la actualidad en gestantes con índice de Bishop desfavorable no se intenta maduración cervical con alternativas potencialmente útiles como el uso de balón cervical o dilatación mediante el uso de sonda Foley transcervical, por lo cual un estudio prospectivo con la aplicación de un modelo predictivo y con la implementación de técnicas de maduración cervical no farmacológicas, permitirían una mejor validación de los modelos.

Teniendo en cuenta el rendimiento similar entre ambos modelos y la facilidad de aplicación del modelo de Metz consideramos este es el de mayor posibilidad de implementación en la mayoría de servicios de obstetricia, sin el requerimiento de acceso a software o programas para la determinación de la probabilidad de éxito de parto vaginal después de cesárea.
